# Supplementary material for: Anxiolytic-like Effects and Quantitative EEG Profile of Palmitone Induces Responses Like Buspirone Rather Than Diazepam as Clinical Drugs
Source: Molecules. 2023 Apr 24;28(9):3680. doi: 10.3390/molecules28093680 (PMC10180017; doi:10.3390/molecules28093680)
Supplement: Supplementary file 1 [file molecules-28-03680-s001.zip › molecules-2280885-supplementary.pdf]

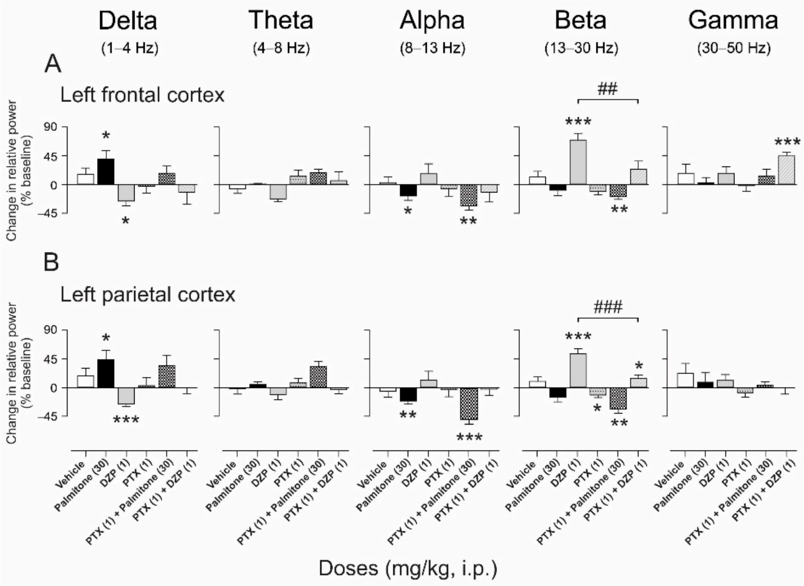


**Figure S1.** Effects of palmitone (30 mg/kg, i.p.) and DZP (1 mg/kg, i.p.) in the presence of the GABAA antagonist PTX (1 mg/kg, i.p.) on the relative power bands of the left frontal (A) and parietal cortex (B). Data are shown as the mean±S.E.M of six repetitions per group. One-sample *t*-tests, *p<0.05, **p<0.01, ***p<0.005 vs. baseline. One-way ANOVA followed by Tukey´s *post hoc* test, ##p<0.00-, ###p<0.0001 DZP vs. PTX + DZP.

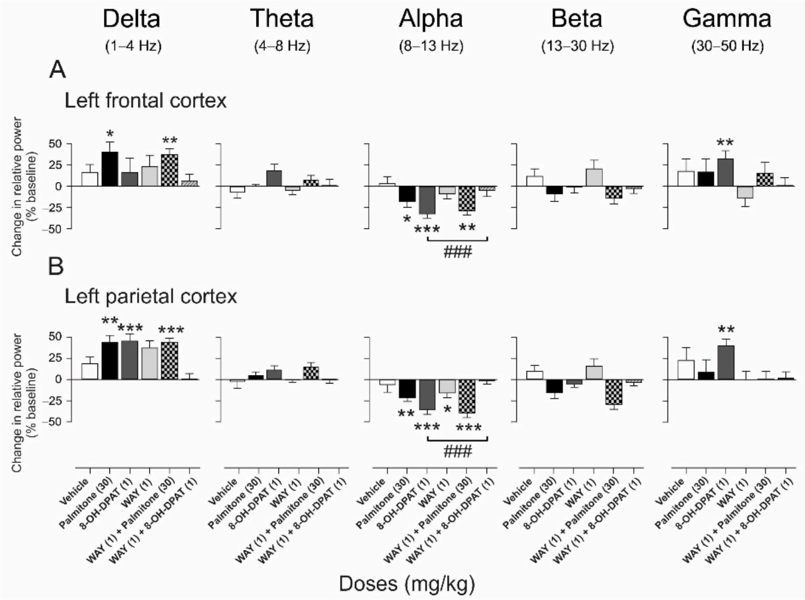


**Figure S2.** Effects of palmitone (30 mg/kg, i.p.) and 8-OH-DPAT (1 mg/kg, s.c.) in the presence of the serotonin 5-HT1A receptor antagonist WAY100635 (WAY, 1 mg/kg, i.p.) on the relative power bands of the left frontal (A) and parietal cortex (B). Data are shown as the mean±S.E.M of six repetitions per group. One-sample *t*-tests, *p<0.05, **p<0.01, ***p<0.005 vs. baseline. One-way ANOVA followed by Tukey´s *post hoc* test, ###p<0.0001 8-OH-DPAT vs. WAY + 8-OH-DPAT.
